# Updated protein domain annotation of the PARP protein family sheds new light on biological function

**DOI:** 10.1093/nar/gkad514

**Published:** 2023-06-16

**Authors:** Marcin J Suskiewicz, Deeksha Munnur, Øyvind Strømland, Ji-Chun Yang, Laura E Easton, Chatrin Chatrin, Kang Zhu, Domagoj Baretić, Stéphane Goffinont, Marion Schuller, Wing-Fung Wu, Jonathan M Elkins, Dragana Ahel, Sumana Sanyal, David Neuhaus, Ivan Ahel

**Affiliations:** Centre de Biophysique Moléculaire, CNRS UPR 4301, Orléans, France; Sir William Dunn School of Pathology, University of Oxford, Oxford OX1 3RE, UK; Sir William Dunn School of Pathology, University of Oxford, Oxford OX1 3RE, UK; Department of Biomedicine, University of Bergen, Bergen, Norway; MRC Laboratory of Molecular Biology, Francis Crick Avenue, Cambridge CB2 0QH, UK; MRC Laboratory of Molecular Biology, Francis Crick Avenue, Cambridge CB2 0QH, UK; Sir William Dunn School of Pathology, University of Oxford, Oxford OX1 3RE, UK; Sir William Dunn School of Pathology, University of Oxford, Oxford OX1 3RE, UK; Sir William Dunn School of Pathology, University of Oxford, Oxford OX1 3RE, UK; Centre de Biophysique Moléculaire, CNRS UPR 4301, Orléans, France; Sir William Dunn School of Pathology, University of Oxford, Oxford OX1 3RE, UK; MRC Laboratory of Molecular Biology, Francis Crick Avenue, Cambridge CB2 0QH, UK; Centre for Medicines Discovery, University of Oxford, Oxford OX3 7DQ, UK; Sir William Dunn School of Pathology, University of Oxford, Oxford OX1 3RE, UK; Sir William Dunn School of Pathology, University of Oxford, Oxford OX1 3RE, UK; MRC Laboratory of Molecular Biology, Francis Crick Avenue, Cambridge CB2 0QH, UK; Sir William Dunn School of Pathology, University of Oxford, Oxford OX1 3RE, UK

## Abstract

AlphaFold2 and related computational tools have greatly aided studies of structural biology through their ability to accurately predict protein structures. In the present work, we explored AF2 structural models of the 17 canonical members of the human PARP protein family and supplemented this analysis with new experiments and an overview of recent published data. PARP proteins are typically involved in the modification of proteins and nucleic acids through mono or poly(ADP-ribosyl)ation, but this function can be modulated by the presence of various auxiliary protein domains. Our analysis provides a comprehensive view of the structured domains and long intrinsically disordered regions within human PARPs, offering a revised basis for understanding the function of these proteins. Among other functional insights, the study provides a model of PARP1 domain dynamics in the DNA-free and DNA-bound states and enhances the connection between ADP-ribosylation and RNA biology and between ADP-ribosylation and ubiquitin-like modifications by predicting putative RNA-binding domains and E2-related RWD domains in certain PARPs. In line with the bioinformatic analysis, we demonstrate for the first time PARP14’s RNA-binding capability and RNA ADP-ribosylation activity *in vitro*. While our insights align with existing experimental data and are probably accurate, they need further validation through experiments.

## Graphical Abstract

**Graphical Abstract:**
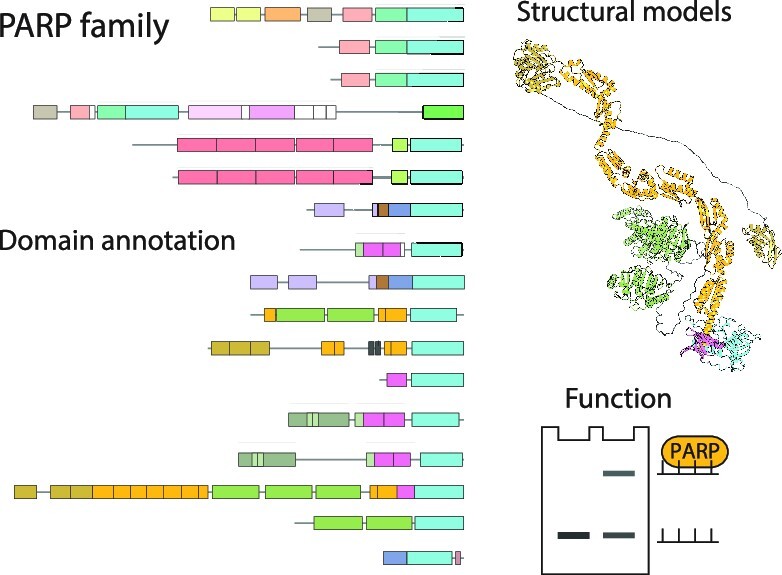

## INTRODUCTION

The human PARP family plays a critical role in ADP-ribosylation signalling, regulating various cellular functions in humans and other higher eukaryotes. In recent years, the ADP-ribosylation field has broadened its focus beyond a few well-studied members to explore the properties and functions of numerous PARP proteins with a different domain composition. Therefore, it is now timely to revisit the domain annotation of the PARP family to establish a better foundation for further development in this field. Recent developments in computational tools, such as AlphaFold2 (AF2), have facilitated this task, as we will discuss below.

Eukaryotic proteins typically comprise multiple structured domains, i.e. distinct structural units with a relatively rigid 3D form (1). During evolution, domains often behave as portable modules with distinct, dedicated functions, such as catalysing a particular chemical reaction or binding to a specific ligand class. Therefore, domain annotation reveals a functional ‘toolkit’ at the disposal of a given protein. Moreover, even in cases where a domain does not have a clearly conserved role, its identification can hint at the biological function of a protein by demonstrating an evolutionary relationship to better characterised proteins that possess the same domain.

Domain annotation has been an important tool in exploring the PARP protein family, which comprises proteins that contain an ADP-ribosyl transferase (ART) domain closely related to that of the founding member, PARP1 (for poly(ADP-ribose) polymerase 1). PARP1, a highly abundant nuclear protein found in animals, was first identified and characterised biochemically (2–6). This revealed its ability to catalyse poly(ADP-ribosyl)ation (PARylation), i.e. covalent connection of ADP-ribosyl units into poly(ADP-ribosyl) (PAR) chains. PAR chains are typically linked to proteins and function as a protein post-translational modification (PTM). PARP1’s catalytic output depends on its C-terminal ART domain, which contains a binding pocket for NAD^+^, the donor of the ADP-ribosyl moiety. The ART domain of PARP1 is distantly related to that of bacterial toxins that modify proteins with single ADP-ribose units (mono(ADP-ribosyl)ation or MARylation) (7). Following the cloning of the human *PARP1* gene (8,9), other proteins with homology to its catalytic part have been identified (10). This discovery led to the eventual description of the PARP protein family (11), which now includes 17 different canonical members (each encoded by a separate gene) in humans (12,13). While some PARP-family proteins might be catalytically inactive, most have been shown to catalyse protein ADP-ribosylation, either MARylation or PARylation (14), and some also ADP-ribosylation of nucleic acids (15). PARPs are typically large and comprise diverse domains besides the signature PARP-type ART domain, hinting at their different specialised functions (11,12,16,17).

Traditionally, protein domains (considered primarily as signature sequence motifs) have been identified based on sequence homology to known instances in other proteins (18). This approach has also been used to define and preliminarily characterise the PARP family (11). Sequence-based approaches benefitted from the development of hidden Markov model (HMM)-based strategies (19) and were more recently supplemented with the analysis of similarity on the secondary structural level, as in the HHPred tool (20–22). However, even sophisticated methods of this kind are imperfect at detecting highly diverged homologues of known domains. As tertiary protein structure generally persists longer in evolution than protein sequence (23–25), approaches that access three-dimensional, tertiary structural information allow more exhaustive detection of homology to known domains. Indeed, the PARP catalytic domain had not been definitively annotated as an ART domain homologous to that in MARylating bacterial toxins until the relevant structures were solved (7). Structural analysis can also more conclusively define new domain types, even though they can be proposed based on sequence analysis alone.

Traditionally, tertiary protein structure has been conclusively resolved only with experimental approaches, applied on a one-by-one basis to individual domains or larger protein fragments. In that respect, the most studied members of the PARP family are PARP1, its close homologue PARP2 and tankyrases (TNKS1/PARP5A and TNKS2/PARP5B). All structured domains of PARP1 have been characterised using X-ray crystallography or nuclear magnetic resonance (NMR) (26–30), whereas for PARP2 full-length cryo-electron microscopy (cryo-EM) and crystallographic structures, with most of its length resolved, are available (31,32). For tankyrases, partial structures of various segments exist (33–38), notably including a recent cryo-EM structure of a noncovalent polymer formed by the C-terminal fragment of TNKS2 (39). For other PARP family members, the available experimental structural information is at best partial.

Recently, experimental approaches to studying protein structure have been supplemented with highly efficient artificial intelligence (AI) prediction systems including AlphaFold—especially its AF2 release (40)—and its analogues or derivatives, including RoseTTAFold (41). These tools can rapidly generate three-dimensional structural models with high accuracy based only on amino-acid sequence as input, allowing faster analysis of whole protein sets. The analysis is generally efficient even for structured protein regions that do not bear detectable similarity to any previously solved structures (40). AF2 utilises two sources of information: experimentally-determined protein structures, deposited in the Protein Data Bank (PDB), which serve as a training set of possible spatial arrangements, and a multiple sequence alignment of the query protein, which allows detection of evolutionary relationships between different segments of the sequence that reflect their spatial proximity. The computational advances that allow AF2 to mine this information efficiently are elegantly explained in a recent article (42). The final AF2 model, produced without any consideration for physicochemical forces, can be relaxed using the Amber force field (40,43). AF2, especially in its Multimer version (44), can also be used for predicting structures of protein complexes, which is generally less accurate than single structure prediction, but works well for stable and evolutionarily conserved interactions. Reportedly, the latest Multimer 3 version is markedly more efficient than the initial protocol. The release of AF2’s code following its outstanding performance at the 14th edition of the Critical Assessment of protein Structure Prediction (CASP) competition has sparked ongoing development of further improvements (45), as evidenced by the result of the most current CASP15 edition.

The new approaches have been made accessible to structural biologists that are not fluent in computational techniques through easy-to-access online resources. One such resource is the AF Protein Structure Database (46) (https://www.alphafold.ebi.ac.uk), an on-line database of precalculated AF2 models of most Swiss-Prot- and UniProt-deposited proteins, generally excluding only very long sequences of >2700 amino-acid residues. Another online platform, ColabFold (47) (https://www.colabfold.com), allows predicting structure from sequence using an optimised AF2-based approach. Prediction of protein complexes through ColabFold is possible by inputting multiple sequences separated by a colon. A further example is provided by FoldSeek (48), an online tool that allows rapid searches for structural homologues of a structure of interest among experimental and predicted structures (https://search.foldseek.com/search). The 3Di/AA (three-dimensional interactions per amino-acid residue) search algorithm used by FoldSeek focuses on local rather than global structural homology, which is advantageous if the relative orientation of more distantly separated elements is inaccurately predicted or has considerably diverged during evolution. Of note, FoldSeek scans only the proteins for which precalculated models are available in the AF Protein Structure Database, thus excluding some extremely long proteins. As an alternative to FoldSeek, combining an older fold recognition tool, DALI (49) (http://ekhidna2.biocenter.helsinki.fi/dali/), with AF2 models has been proposed (50).

**Figure 1.**
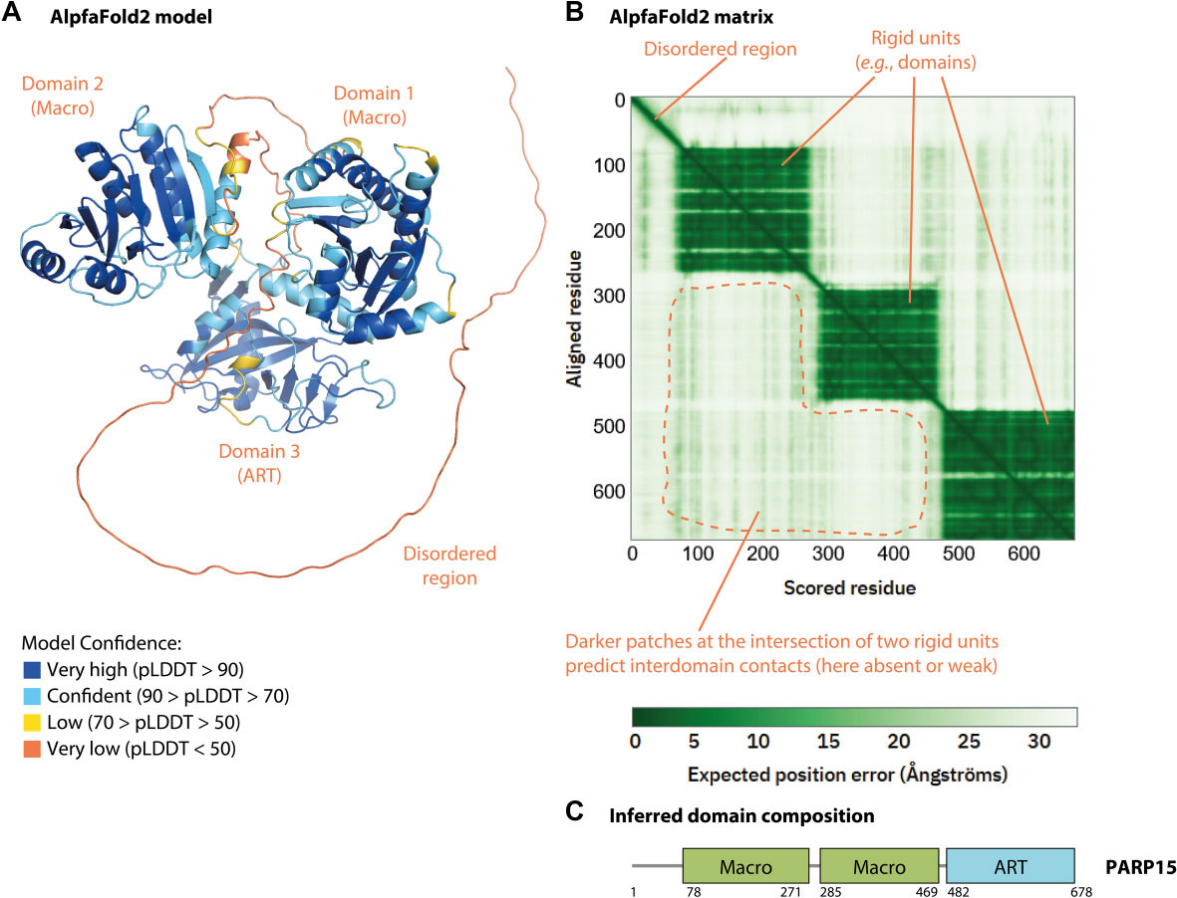
Interpretation of AlphaFold2 results. (A and B) A typical AlphaFold2 result including a model (**A**) and matrix (**B**) taken from the AlphaFold Protein Structure Database. The results for human PARP15 (PAR15_HUMAN) are shown. The images are annotated in orange. pLDDT stands for predicted local distance difference test, a per-residue confidence score calculated by AlphaFold (between 0 and 100). In (B) and in subsequent similar cases, we highlight, using a dotted orange line, regions of the matrix corresponding to relative domain immobilisation (indicative of interdomain contacts) only below the diagonal, while omitting, for the sake of clarity, a quasi-symmetrical equivalent region above the diagonal. (**C**) The inferred domain diagram of PARP15—aligned with the matrix. The matrix indicates that the three domains are flexible relative to each other.

Despite being developed only recently, AF2 and related methods have already triggered a revolution in protein science by allowing highly accurate prediction of structured and unstructured (intrinsically disordered) regions in proteins, and of the fold of structured parts (51). The default AF2 output includes i) a structural model of a protein and ii) a matrix showing the expected relative positional error of residues within the sequence (40,52). When run with standard settings, ColabFold generates five models and corresponding matrices for each sequence, which allows an assessment of the reproducibility of prediction. In a default presentation, the structural model is coloured according to confidence scores (provided in the B-factor column in the output PDB file), in which structured domains typically correspond to high-confidence regions, whereas intrinsically disordered segments are represented as low-confidence regions (Figure 1A). The AF2 matrix provides another representation of the data, allowing identification of segments that form rigid units (corresponding to structured domains or their rigid assemblies), which manifest as dark green rectangles, and intrinsically disordered regions, which produce dark green lines (Figure 1B). We can infer the domain arrangement from these outputs (Figure 1C). Additionally, the matrix can help prediction of inter-domain interactions (in shades of green) that could partially immobilise some domains relative to each other, as will be illustrated later.

Here, we used AF2 models, as well as some recent experimental studies, to update the domain composition of the human PARP family members relative to the annotation contained in the Pfam (53) and InterPro (54) databases and the available literature. Owing to the unprecedented accuracy of the new algorithms, this annotation is expected, for the first time, to be essentially complete, at least with respect to all larger structured domains and long intrinsically disordered regions. Among other insights, our analysis predicts a previously unknown prevalence of K homology (KH) domains in a subset of PARPs. These domains could mediate sequence-specific RNA binding. Additionally, we offer some insights into the overall structure of individual PARP proteins, probable interdomain contacts, and provide some functional interpretation of our observations. The presented predictions—while consistent with available experimental data and likely to be highly accurate—remain to be verified and extended using experimental approaches. In addition, both by performing NMR analysis of PARP1 and through biochemical tests of PARP14’s RNA-binding capability and RNA ADP-ribosylation activity, we show how experiments can complement AF2 predictions.

## MATERIALS AND METHODS

### Protein sequences

Standard sequences of isoforms 1 of 17 human PARPs were retrieved from UniProtKB reviewed entries with the following names: PARP1_HUMAN, PARP2_HUMAN, PARP3_HUMAN, PARP4_HUMAN, TNKS1_HUMAN, TNKS2_HUMAN, PARPT_HUMAN (for PARP7/TIPARP), PARP8_HUMAN, PARP9_HUMAN, PAR10_HUMAN, PAR11_HUMAN, PAR12_HUMAN, ZCCHV_HUMAN (for PARP13/ZAP), PAR14_HUMAN, PAR15_HUMAN, PAR16_HUMAN.

### Structural model analysis

AF2 structural models were retrieved from the AF Protein Structure Database (40,46) (https://www.alphafold.ebi.ac.uk, version 2022-11-01, created with the AlphaFold Monomer v2.0 pipeline). Structural models and corresponding matrices were analysed using the database online interface, and, in the case of the models, additionally in PyMol, which was used for structural figure preparation. Where structures are coloured according to confidence, colours have been set as defined by Konstantin Korotkov. Structural alignments with available experimental structures were performed in PyMol using the ‘super’ command with selected protein fragments. RMSDs were calculated over C_alpha_ atoms only (specified using ‘name ca’ in PyMol).

### FoldSeek

FoldSeek (48) (https://search.foldseek.com/search) analysis was performed using PDBs of isolated domains as input and standard settings (all databases available on 15/12/2022, mode 3Di/AA).

### Hhpred

HHPRED (20,21) (https://toolkit.tuebingen.mpg.de/tools/hhpred) analysis was performed using the HHPRED section of the Max Planck Institute (MPI) Bioinformatic Toolkit (22) with full-length or truncated protein sequences as input and standard settings, including the ‘PDB_mmCIF70_12_Aug’ database.

### ConSurf

ConSurf (65) (https://consurf.tau.ac.il, version updated in 2019) analysis was performed using the PDB of the isolated MVPID domain (residues 1570–1724) from the AF2 model of human PARP4 with standard settings.

### Protein expression and purification for NMR analysis

DNA coding for human PARP1 (residues 2–1014) containing the V762A point mutation was amplified by PCR from a codon-optimised human *PARP1* gene (Qiagen), and subcloned into a pET28a vector using XbaI and XhoI restriction sites to carry an N-terminal His_6_ tag (MKHHHHHHKMQ). Full expression was performed in the presence of 10 mM benzamide and 35 μg/ml kanamycin. The plasmid was transformed into *Escherichia coli* BL21 (DE3) cells (Stratagene); resulting colonies were resuspended in LB medium containing 35 μg/ml kanamycin and the cells grown at 37°C, 200 rpm until a cell density of ∼2 *A*_600_ was obtained. The culture was diluted 1:40 in M9 minimal medium containing 35 μg/ml kanamycin and 10mM benzamide, and supplemented with ^15^NH_4_Cl (1 g/L) (Sigma Aldrich Isotec) as the sole nitrogen source. The cells were grown at 37°C, 200 rpm until a cell density of 0.8–1.2 *A*_600_ was achieved before arresting cell growth by incubating at 2–8°C for 1 hour. Protein expression was induced by adding 0.5 mM IPTG, supplementing with 0.1 mM ZnSO_4_ and incubating at 25°C, 200 rpm for 18 h. Cells were harvested by centrifugation, resuspended in 25 mM HEPES-Na, pH 8.0, 0.5 M NaCl, 0.5 mM TCEP, 1 mM PMSF and protease inhibitor mix (Roche Complete Protease Inhibitor Cocktail EDTA free; 1 tablet per 50 ml), lysed by sonication and clarified by centrifugation. The clarified harvest was filtered (0.22 μm PVDF Stericup, Millipore), and purified on a 5 ml HisTrap FF (Cytiva) eluting with a linear imidazole gradient in 50 mM HEPES, pH 7.5, 0.5M NaCl, 0.5 mM TCEP. The eluted protein was diluted from 500 to 375 mM NaCl using 50 mM Tris, 0.5 mM TCEP, pH 7.0, and further purified using 5 ml HiTrap heparin FF (Cytiva), eluting with a linear NaCl gradient in 50 mM Tris, 0.5 mM TCEP, pH 7.0. The purified protein was then buffer exchanged into 10 mM sodium phosphate, 5% ^2^H_2_O, 222 mM KCl, 2 mM [^2^H_10_]-DTT, pH 7.4 using a 50 KDa MWCO Vivaspin 20 (Sigma-Aldrich).

*PARP1* BRCT domain (residues 383–525) was subcloned into the pET28a-lip vector; the resulting plasmid contains the sequence for N-terminally His6-tagged *Geobacillus stearothermophilus* di-hydrolipoamide acetyltransferase (UniProt P11961) lipoyl-binding domain, followed by a TEV cleavage site, followed by the sequence for PARP1(383–525). Protein was expressed and purified essentially as described previously for the uniformly [^2^H,^13^C,^15^N] labelled ZnF1–ZnF2–ZnF3 and WGR fragments of PARP1 (30), except that deuterium incorporation was not used (normal H_2_O was used rather than ^2^H_2_O, and [^13^C_6_]-glucose was used rather than [^2^H_7_,^13^C_6_]-glucose).

### DNA dumbbell ligand for NMR analysis

The 45 nucleotide DNA dumbbell ligand (sequence 5′ P GCTGGCTTCGTAAGAAGCCAGCTCGCGGTCAGCTTGCTGACCGCG 3′) was obtained by chemical synthesis (Integrated DNA Technologies Inc.) and purified as described previously (30).

### NMR spectroscopy

All NMR measurements employed in-house Bruker DMX 600 MHz or Avance III HD 800 MHz spectrometers equipped with 5 mm [^1^H,^13^C,^15^N]-cryogenic probes. NMR samples of full-length PARP1 and its complex with the DNA dumbbell were prepared and measured in 10 mM sodium phosphate, 222 mM sodium chloride, 2.7 mM potassium chloride and 2 mM [^2^H_10_]-DTT in 95:5 H_2_O/^2^H_2_O at pH 7.4; extensive testing revealed that under these conditions both the full-length free protein and its complex with the dumbbell DNA remained soluble at least overnight at the concentrations employed for the NMR measurements. Protein concentration for both the free and DNA-bound samples was 35.5 μM. Both were made up separately from the same freshly prepared protein solution stock; the complex was formed by careful addition of protein into a more concentrated DNA solution to reach a final slight excess of DNA (40 μM, 1:1.13). TROSY spectra were obtained at 800 MHz and 25°C using shaped sample tubes (Bruker BioSpin GmbH) designed to maximise sensitivity for lossy samples; intensities in Figure 3 were adjusted for small differences in the acquisitions (free protein, NS = 1102, approx. 34h expt. time; DNA-bound protein, NS = 1296, approx. 38h expt. time). NMR samples of BRCT domain (PARP1 383–525; includes part of the C-terminal linker) were prepared in 50 mM [^2^H_11_] Tris.HCl, 200 mM NaCl, 100 μM ZnSO_4_, 4 mM [^2^H_10_]-DTT, 0.02% NaN_3_ and 0.2 × Roche EDTA-free Complete protease inhibitors in 95:5 H_2_O/^2^H_2_O at pH 7.0. All experiments were conducted at 25°C, and ^1^H chemical shifts were calibrated using sodium 3,3,3-trimethylsilylpropionate (TSP) as an external ^1^H reference; ^15^N and ^13^C chemical shifts were indirectly referenced to the ^1^H shifts using the ratio of gyromagnetic ratios (127). The following datasets were acquired for ^15^N,^13^C-labelled PARP1 BRCT domain to make an essentially complete set of backbone assignments: 2D: [^15^N–^1^H] HSQC and constant-time [^13^C–^1^H] HSQC covering only the aliphatic ^13^C region; 3D: CBCANH, CBCA(CO)NH, HBHANH and HBHA(CO)NH. Partial amide group assignments for full-length PARP1 in both the free and DNA-bound states were made by careful comparisons with fully assigned spectra of separate domains or fragments of PARP1 recorded during previously published projects, specifically ZnF1–ZnF2 (F1F2) and Zn1–Zn2–Zn3 (F1F2F3) (30), BRCT (this work), WGR (30), and CAT (125). NMR data were processed using the programs TopSpin 3.2 or 3.5 (Bruker BioSpin GmbH) and analysed using the programs CcpNMR Analysis 2.4.2 (128) or Sparky version 3.115. (129).

### Cyanine3-labelled DNA and RNA oligonucleotides

The Cyanine3 (Cy3)-labelled RNA and DNA oligonucleotides were acquired from Merck and are listed in Table 1. The oligos were dissolved in 20 mM HEPES–KOH (pH 7.6) and 50 mM KCl.

**Table 1.** Oligonucleotides used for RNA binding and RNA/DNA ADP-ribosylation assays

Oligonucleotide	Sequence 5′-3′
E21 DNA	GTGGCGCGGAGACTTAGAGAA[Cy3]
5′P E21 DNA	[Phos]GTGGCGCGGAGACTTAGAGAA[Cy3]
3′P E21 DNA	[Cy3]GTGGCGCGGAGACTTAGAGAA[Phos]
E21 RNA	[Cy3]GUGGCGCGGAGACUUAGAGAA
5′P E21 RNA	[Phos]GUGGCGCGGAGACUUAGAGAA[Cy3]
3′P E21 RNA	[Cy3]GUGGCGCGGAGACUUAGAGAA[Phos]

### ADP-ribosylation assay with cyanine3-labelled RNA or DNA oligonucleotides

ADP-ribosylation of Cy3-labelled DNA and RNA oligonucleotides was performed as described earlier (101,130). Proteins for the assay were purified as described in the same studies. Briefly, 10 μL reactions were prepared in ADP-ribosylation buffer (20 mM HEPES–KOH (pH 7.6), 5 mM MgCl_2_ and 1 mM DTT). The reactions contained 1 μM Cy3-labelled RNA or DNA oligonucleotide, 3 μM PARPs, PARP10 ART (residues 868–1025), or PARP14 WWE-ART (residues 1459–1801), and 500 μM NAD^+^. The reactions were incubated for 1 h at 37°C and stopped by adding 50 ng/μl Proteinase K and 0.15% SDS followed by incubating at 50°C for 30 min. Finally, the reactions were mixed with 2× TBE urea sample buffer (8 M urea, 20 μM EDTA (pH 8.0), 20 μM Tris–HCl (pH 7.5), and bromophenol blue) and loaded on a pre-run 15% denaturing urea polyacrylamide gel electrophoresis (PAGE) gel. The gels were run at 7 W/gel and imaged using the Molecular Imager PharosFX system (BioRad) with laser excitation for Cy3 at 532 nm.

### Protein expression and purification for the RNA binding assay

PARP14 WWE-ART was prepared as described previously (101), whereas PARP14 KH1-KH2 (residues 316–468) and K8-WWE-ART (residues 1453–1801) were generated in the current studies. First, plasmids expressing adequate fragments of the codon-optimised human *PARP14* gene were cloned into a pET-28a vector using BamHI and XhoI restriction sites. These plasmids were transformed into Rosetta (DE3) competent cells and grown in 2× YT media supplemented with kanamycin and chloramophenicol. Induction was carried out at 0.6–0.8 OD_600_ using 0.5 mM IPTG and cells were allowed to grow overnight at 18°C. Bacterial pellets were lysed in lysis buffer (20 mM HEPES pH 8, 500 mM NaCl, 10 mM imidazole, and 0.5 mM TCEP) supplemented with BugBuster (Merck Millipore), cOmplete EDTA-free protease inhibitor cocktail (Roche), benzonase, and lysozyme. Cleared lysate was applied to a pre-washed Ni-NTA agarose resin followed by washes with lysis buffer. Proteins were eluted using elution buffer (20 mM HEPES pH 8, 500 mM NaCl and 0.5 mM TCEP) with an incremental gradient of 10–500 mM imidazole. Proteins purity was assessed by sodium dodecyl sulphate (SDS)-PAGE gel and further dialysed overnight against 20 mM HEPES pH 7.5, 150 mM NaCl and 0.5 mM TCEP.

### RNA binding (electrophoretic mobility shift) assay with cyanine3-labelled RNA oligonucleotides

Binding reactions were prepared in EMSA buffer (20 mM HEPES–KOH (pH 7.6), 5 mM MgCl_2_, 1 mM DTT, and 20% v/v glycerol). The reactions contained 0.5 μM E21 RNA and 1 μM, 3 μM, or 5 μM of the target proteins PARP14 KH1–KH2 (residues 316–468), PARP14 KH8-ART (residues 1453–1801) and PARP14 WWE-ART (residues 1459–1801). The reactions were incubated for 1 h at room temperature and then loaded on a pre-run 6% native PAGE gel and run at 10 V/cm for 1 h. The gels were imaged using the Molecular Imager PharosFX system (BioRad) with laser excitation for Cy3 at 532 nm.

## RESULTS

### General remarks

We scrutinised AF2 structural models and corresponding matrices, deposited in the AF Protein Structure Database, of the human proteins that comprise the PARP protein family. In the core part of our study we focused on 17 canonical human PARPs as defined by the recent community consensus (13). Additionally, as described in the last section of the paper, we used FoldSeek to search for other proteins whose ART domains are structurally highly similar to the ART domain of PARP1 that could potentially be included as new members of the PARP family in humans.

**Figure 2.**
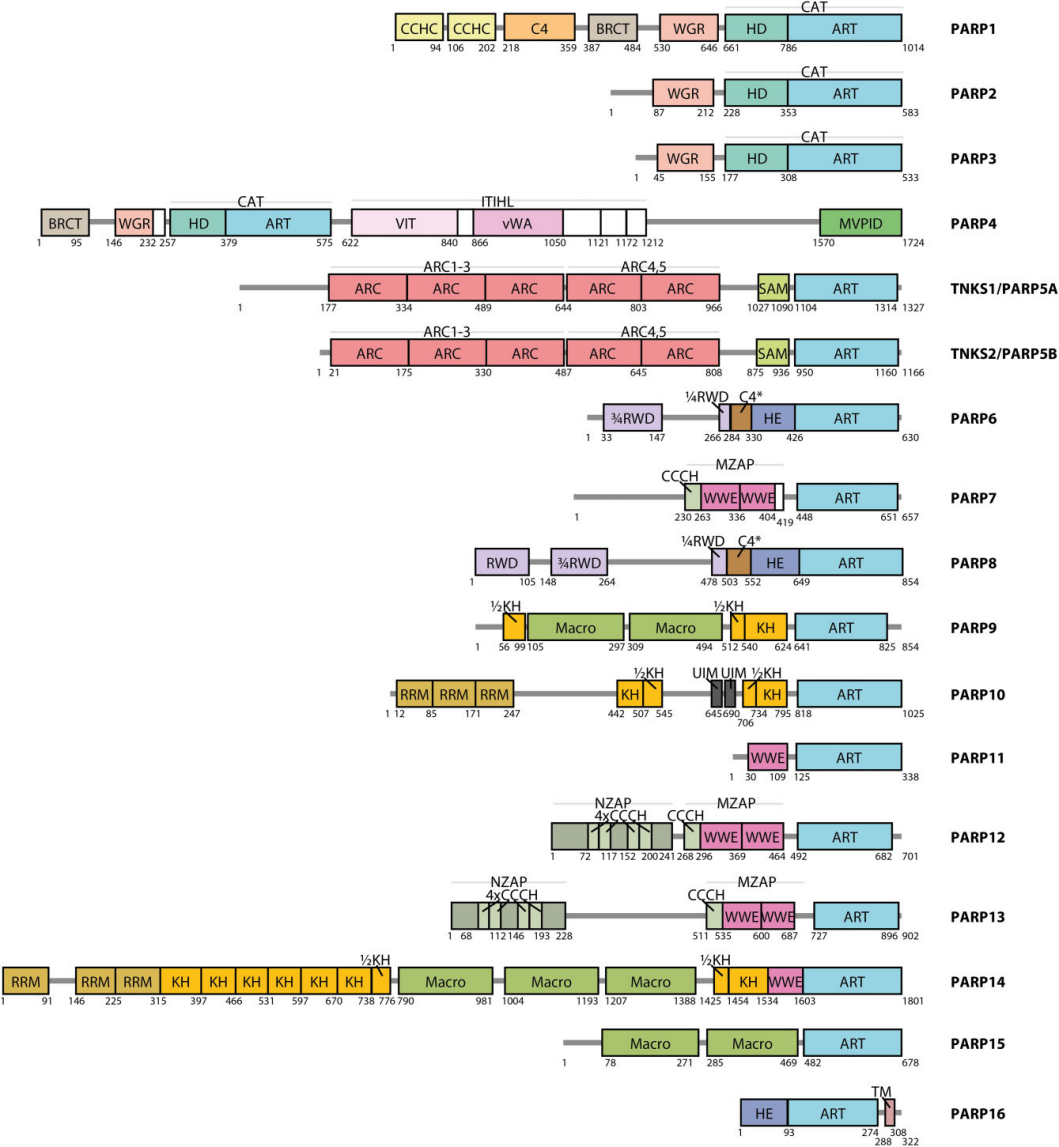
Domain architecture of the human members of the PARP protein family. Domains and other longer structured elements are indicated as boxes and labelled with the domain or motif name. Some structured elements (subdomains, connectors) are shown as unlabelled white boxes. Some larger rigid arrangements composed of multiple subdomains are additionally indicated with light grey lines and labelled above the boxes. Disordered regions are shown as dark grey lines. Residue numbers corresponding to approximate domain or motif boundaries are indicated below. The details of the underlying analysis and definitions of all abbreviations are provided in the main text or in Materials and Methods.

The results of our analysis of canonical PARPs are summarised in an updated overview of the domain architecture of the PARP family (Figure 2), in which the grey line corresponds to regions largely devoid of structure and rectangular boxes to structured domains or motifs. Previously unannotated domains were identified visually or by performing structural searches with the FoldSeek server. Domain identity was then confirmed by visual inspection of structural alignments with available experimental structures of the identified domains. Approximate domain boundaries were estimated based on the models and, where possible, verified using experimental structural information. Most structured regions are labelled with a domain or motif name, but some structured extensions or connectors between domains are shown unlabelled as white boxes. As a rule, we did not annotate short isolated elements with residual secondary structure propensity predicted by AF2, some of which could correspond to binding sites that become folded upon binding to other proteins; the only exceptions of this kind are two ubiquitin-interacting motifs (UIMs) with helical propensity in PARP10, which we annotated based on past experimental confirmation (55). Reserving the term ‘domain’ for structured modules, we did not mark on the scheme intrinsically disordered regions that have previously been given the name ‘domain’ (e.g. His-, Pro- and Ser-rich (HPS) domain of TNKS1).

Two domains in our scheme—namely the helical domain at the very C terminus of PARP4 (residues 1570–1724) and the C4-type ZnF in PARP6 (residues 284–330) and PARP8 (residues 503–552)—do not bear any homology to previously characterised domains in any other human protein. Since the C-terminal segment of PARP4 corresponds to the previously reported interaction site for the major vault protein (MVP), we propose, partially following previous practice (56), to use the name MVPID (for ‘MVP-interacting domain’).

In cases where multiple subdomains come together to form a larger structural arrangement—as for example in the case of the inter-alpha-trypsin heavy chain (ITIH)-like (ITIHL) region of PARP4—we label the whole region above the boxes, while also keeping labels of individual boxes corresponding to more distinct subdomains. Subdomains that are integral to the larger arrangement are not necessarily labelled with a distinct name and are shown as white boxes.

Among previously unannotated domains, we have identified instances of ‘split’ domains (of the KH and RWD class, the latter related to ubiquitin-conjugating enzymes, E2), which are contiguous in structure but, at the sequence level, comprise two parts separated by a long insertion. Such split domains are indicated in Figure 2 using approximate fractions (e.g. ‘¾RWD’ and ‘¼RWD’ to indicate two parts of an RWD in PARP6 and PARP8). The insertions within such split domains are seemingly either mostly intrinsically disordered (in PARP6 and PARP8) or include other motifs or domains flanked by disordered linkers (as in PARP9, PARP10, PARP14); the most striking example of the latter case is a long insertion with three Macro domains within a predicted split KH domain in PARP14.

When producing the updated scheme, we tried to prevent confusion that was generated previously by the use of the same names for domains that are only superficially similar. Thus, we keep the name HD (‘helical domain’) only for the characteristic regulatory helical subdomain identified in PARP1 and also conserved in PARP2, PARP3 and PARP4. In these proteins, the HD and the ART subdomains together constitute the CAT (catalytic) domain. In contrast, we propose the name HE (for ‘helical extension’) for a structurally different all-helical appendage to the ART domain observed in PARP6, PARP8 and PARP16. Similarly, we differentiate between different types of zinc-fingers (ZnFs) in PARPs, referring to them according to the residues that coordinate zinc (CCHC, C4 or CCCH), which in all these cases goes hand in hand with a different overall structure of these motifs. Since there are two structurally different C4 ZnFs in PARPs, we refer to the one in PARP6 and PARP8 as C4*, to distinguish it from the third ZnF of PARP1.

Importantly, AF2 models appear to represent a protein without ligands such as DNA, RNA, or small molecules, but in reality they might reflect conformations that are only sampled in the presence of ligands. This is because the evolutionary relationships between different protein regions that AF2 detects based on a multiple sequence alignment and uses for prediction have been shaped in the biological milieu where various potential ligands important for function are present. Moreover, AF2 results might be partially influenced by previous experimental structures solved in the presence of ligands. We illustrate this problem by comparing the AF2 matrix for PARP1—which indicates possible rigidifying interactions between different PARP1 domains—with an NMR analysis of PARP1 domain flexibility in the presence and absence of DNA, which suggests that different PARP1 domains (except for the BRCT domain) become immobile relative to each other only upon binding to a DNA break.

Below, we use root-mean-square deviation (RMSD) between models and experimental structures of related domains as an estimate of potential evolutionary relatedness or divergence. However, while the generally high accuracy of AF2 models suggests that such an interpretation is likely often to be justified, it should be born in mind that, if there are cases in which an AF2 model is inaccurate, these would also lead to observed deviations. We therefore caution the reader that the RMSD-value comparisons are contingent on the compared models being accurate to a similar extent.

Below, we offer a detailed description of individual PARPs clustered together into small groups according to structural and evolutionary similarity. Our clustering overlaps with division of the family into clades (57,58), except that we have subdivided the heterogenous clade 3 into subgroups.

### PARP1, PARP2 and PARP3

DNA repair-associated PARPs (also known as clade 1)—PARP1, PARP2 and PARP3—have been extensively studied with experimental methods and AF2 models do not bring considerable new knowledge about their domain composition. Moreover, the case of these PARPs demonstrates the importance of experimental approaches, which are so far indispensable for studying phenomena such as interaction with non-protein ligands (nucleic acids, NAD^+^ and its analogues), allostery, conformational diversity and dynamics, all of which appear key to understanding how PARPs function. Having said that, future detailed comparison of AF2 models with specific conformations of these PARPs (e.g. autoinhibited *vs*. active) might illuminate the extent to which AF2 models could assist in the study of dynamic systems.

PARP1 is known to be activated by various forms of DNA damage, which are detected by ZnFs and the WGR domain (named after conserved amino-acid residues), leading to partial displacement and unfolding of the HD subdomain (59). Since, in the DNA-free state, the HD inhibits NAD^+^ access to the PARP active site, its rearrangement (still incompletely characterised at the structural level) in response to DNA damage binding leads to activation of the ADP-ribosylation activity (60). The same allosteric mechanism appears to govern the activation of PARP2 and PARP3, which, in the absence of ZnFs, detect DNA damage solely through the WGR domain.

**Figure 3.**
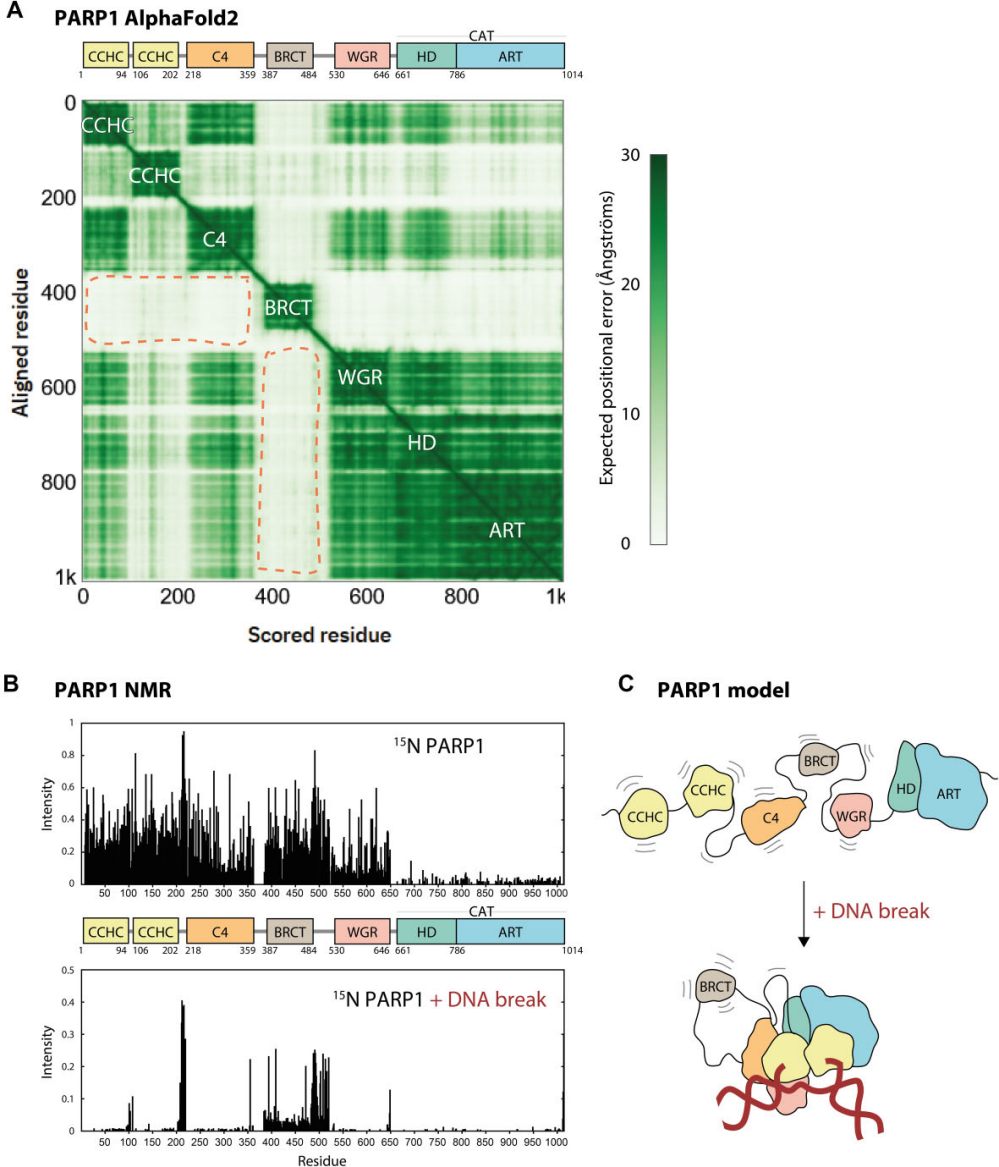
Insights into structure and dynamics of PARP1 from AlphaFold2 models and NMR experiments. (**A**) AlphaFold2 matrix and aligned domain architecture of PARP1. Dark squares corresponding to PARP1 domains are labelled on the matrix. Light rectangles at the intersection of BRCT and other domains (indicated with dashed orange lines) suggest that no stabilising interdomain interactions are made by BRCT. (**B**) Backbone amide signal intensities from [^15^N,^1^H]-TROSY spectra of ^15^N-labelled PARP1 with or without a DNA dumbbell mimicking a single-strand break. (**C**) A model of PARP1 domain mobility in the absence or presence of a DNA break.

PARP1 additionally contains a BRCT (BRCA1 C terminus) domain—flanked by flexible linkers—which has been implicated in interactions with proteins, intact DNA, or PAR (61,62). Of interest is the AF2 matrix of PARP1 (Figure 3A), which suggests that while ZnFs, WGR, HD and ART are largely immobile relative to each other—suggesting stabilisation by interdomain contacts—BRCT remains flexible relative to the rest of the protein, highlighting its independence. To compare these predictions with experimental data, we have carried out NMR analysis, probing [^15^N,^1^H]-TROSY spectra of ^15^N-labelled full-length PARP1 in the presence or absence of a DNA dumbbell ligand that mimics a single-stranded DNA break (Supplementary Figure S1). Subsequently, we quantified the intensity of ^15^N,^1^H crosspeaks along the PARP1 sequence, which gives an indication of domain mobility (Figure 3B). In the absence of DNA, all the small domains in PARP1 (three ZnFs, BRCT, WGR) behave as if they have independent mobility, producing sharp signals and high-intensity crosspeaks, not dissimilar from those that would be seen for the isolated domains. Upon DNA binding, most of the crosspeaks for ZnFs and WGR domains disappear, suggesting that these domains become incorporated into a larger rigid body; the much slower overall tumbling of such a particle results in much broader NMR signals that are essentially undetectable in these experiments. Only the BRCT domain and longer linker regions still show high-intensity cross peaks in the DNA-bound state, suggesting that they retain their independent mobility. Consistent with this, the fact that the BRCT crosspeaks from the sample in the DNA-bound state have essentially unchanged chemical shifts relative to those from the spectrum of either the free full-length protein or of an isolated BRCT domain suggests there are no significant interactions between BRCT and other domains (Supplementary Figure S1). Due to its larger size, signals from the CAT domain (composed of HD and ART) in the free protein were much weaker than those of the other, smaller, domains, in these experiments, precluding a similar assessment of changes in dynamics upon DNA binding for CAT. Overall, these data are consistent with a model whereby PARP1 behaves as beads on a string that collapse into a more rigid structure upon DNA break binding, with only BRCT excluded from the bound arrangement (Figure 3C).

Notably, the AF2 matrix, and indeed the AF2 structural models of PARP1, appear to be more consistent with the DNA-bound state, presumably reflecting the evolutionary importance of the DNA-dependent inter-domain interactions, as well as the fact that the available crystal structures of multi-domain forms of PARP1 are all of DNA-bound states.

### PARP4

The sole human member of clade 5, PARP4 (also known as VPARP for ‘vault PARP’), was first identified as an associated component of vaults, enigmatic ribonucleoprotein structures present in eukaryotic cells (56). The association with these structures was suggested to occur via a C-terminal region, while the N-terminal part was shown to exhibit homology to PARP1, and the central part to the inter-alpha-trypsin protein. The AF2 model confirms this general architecture while providing more detail.

**Figure 4.**
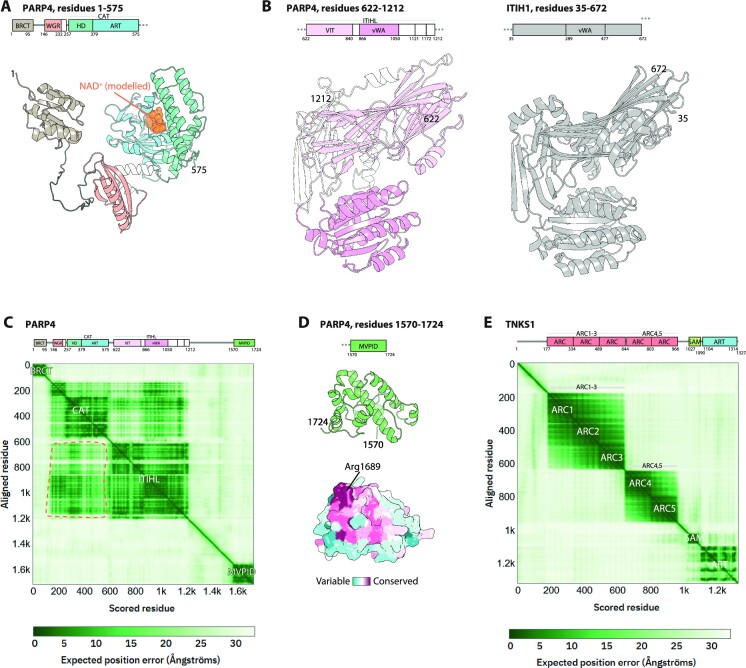
Insights into structure of PARP4 and TNKS1 from AlphaFold2 models. (**A**) Domain architecture and AlphaFold2 structural models of the PARP1-homology fragment of PARP4. NAD^+^ (orange spheres) was modelled in based on the structure of PARP1 ART bound to benzamide adenine dinucleotide (PDB 6BHV). Structural models are coloured according to domain composition (also in panel B). (**B**) Domain architecture and AlphaFold2 structural model of the ITIHL region of PARP4 (left) compared to the crystal structure of ITIH1 (PDB 6FPY, right). (**C**) AlphaFold2 matrix and aligned domain architecture of PARP4, with domains and rigid arrangements of domains labelled on the matrix. The darker patches at the intersection of CAT and ITIHL regions (indicated with dashed orange lines) suggest interdomain interactions. (**D**) AlphaFold2 structural model of the MVPID of PARP4. Both a ribbon and a surface representation are shown, left and right respectively, with the surface coloured according to sequence conservation. The conserved Arg1689 residue is indicated. (**E**) AlphaFold2 matrix and aligned domain architecture of TNKS1.

According to the AF2 model, the N-terminal region (residues 1–575) of PARP4 is very close to PARP1 in domain composition, complete with a BRCT domain, a WGR domain, an HD and the ART (Figure 4A). The WGR and HD domains have not been reported before, although they can be detected with sequence-based HHPred. These two domains in the model superpose well with those in experimental PARP1 structures, with (RMSD) of ∼2 Å over ∼50 core C_alpha_ atoms for the WGR domain and ∼2 Å over ∼100 C_alpha_ atoms for the HD (aligned with the corresponding portions of PDB 4DQY). Overall, similar architecture of this part suggests that PARP4—like PARP1, PARP2 and PARP3—could recognise DNA breaks (or other types of nucleic acid ligands) and be activated in an allosteric manner that involves HD. However, previous failure to detect WGR and HD homology reflects divergence at the sequence level that could indicate altered function. Detailed analysis of the conservation of specific residues might shed more light on this question.

As noticed before (56), the central part of PARP4 is homologous to inter-alpha-trypsin heavy chain (ITIH) found in proteins implicated in, among other roles, modulation of innate immunity (63). In the AF2 model this part of PARP4 corresponds to a large structured arrangement that we propose to term an ITIH-like (ITIHL) region (Figure 4B, left). Recently, the corresponding fragment of the ITIH1 protein—which is close in structure to that in PARP4 over most of its length (RMSD of ∼5 Å over ∼400 C_alpha_ atoms)—has been determined by X-ray crystallography, revealing similarity to integrin (64) (PDB 6FPY) (Figure 4B, right). The ITIHL region of PARP4 includes the previously annotated vault protein inter-alpha-trypsin (VIT) and von Willebrand factor type A (vWA) domains, which are closely packed with each other and other ITIHL elements, making together a large, convoluted structured whole. PARP4’s similarity to ITIH1 and, ultimately, integrin, could suggest binding to some of the same partners, potentially including cell adhesion proteins or complement components (63). However, as these putative partners are generally extracellular, it is not clear if they could be accessible to PARP4. The question of the possible binding partners of the ITIHL region of PARP4 awaits experimental investigation.

Of interest, the AF2 matrix indicates that the PARP1-like N-terminal section of PARP4 forms putative interactions with the ITIHL fragment, hinting at a possible functional connection between these two parts (Figure 4C).

Finally, we looked closely at the C-terminal segment which has been shown to mediate interaction with vaults and named MVP-interacting domain. We propose to abbreviate it to MVPID and use this as a temporary name until the function of this domain is further clarified. The AF2 model predicts that MVPID corresponds to a structured, all-helical domain of around 150 amino acids (Figure 4D). We used the ConSurf server (65) to map sequence conservation across species on the surface of the model, revealing a conserved patch on one side, centred around residue Arg1689. This could correspond to the binding site to MVP or another prominent factor. A structural homology search performed with FoldSeek suggested that the only other human protein with a similar domain (within the sensitivity offered by this tool) is a poorly characterised protein, von Willebrand factor A domain-containing protein 5A (VMA5A). The similarity between the MVPID domain of PARP4 and the equivalent region in the modelled VMA5A structure is moderate (RMSD of ∼6.5 Å over 100 C_alpha_ atoms), and the VMA5A does not contain a residue equivalent to Arg1689, possibly reflecting functional divergence. VMA5A also contains an ITIHL region similar to that in PARP4 (RMSD of ∼5 Å over ∼400 C_alpha_ atoms).

PARP4 has been shown—alongside PARP9, PARP13, PARP14 and PARP15—to undergo rapid evolution that could suggest a role in host-virus rivalry (66,67). The region of PARP4 that had been positively selected in primates was mapped to the area around residues 1504–1521, which might therefore interact with some viral-derived factor (66). In the AF2 model, this region is in an intrinsically disordered segment linking ITIHL to MVPID, suggesting that, in PARP4 from some species, it could be a site of proteolytic cleavage or other post-translational modification (PTM) by viruses, possibly leading to altered association with vaults. Notably, vaults have been implicated in innate immunity and viral infection (68,69).

### Tankyrases (TNKS1/PARP5A and TNKS2/PARP5B)

As tankyrases (clade 4) have been relatively well characterised experimentally, AF2 models do not bring much new insight into their domain organisation, but structure prediction could nonetheless aid in studying some aspects of these proteins. Tankyrases are PARylating enzymes involved in signalling. They are unique among PARPs in containing ankyrin repeat cluster (ARC) domains, which serve as substrate recruitment platforms by recognising specific linear motifs in tankyrase substrates, as illustrated by peptide-bound crystal structures (70). Of note is the AF2 matrix of TNKS1 (Figure 4E) and TNKS2, which indicates two rigid units, one formed by ARC1, ARC2 and ARC3, and the other by ARC4 and ARC5, that can move relative to each other. This is consistent with experimental data obtained for TNKS1 by small-angle X-ray scattering (SAXS) (37).

In addition to ARCs and the C-terminal ADP-ribosyl transferase (ART) domain, tankyrases contain a sterile alpha motif (SAM) domain that is known to mediate formation of noncovalent helical filaments (head-to-tail polymers) (35,36). The recent analysis of the SAM-ART portion of TNKS2 showed that polymerisation driven by SAM leads to contacts between neighbouring ART domains in a chain that appear important for full catalytic activity (39).

Lastly, AF2 models recapitulate the previously reported integrated CHCC ZnF within the ART domain of both tankyrases (residues 1232–1246 in TNKS1 (33)). As this ZnF originates from a transformed loop of the ART and does not constitute a distinct subdomain, we did not indicate it in Figure 2.

### PARP6, PARP8, PARP16

**Figure 5.**
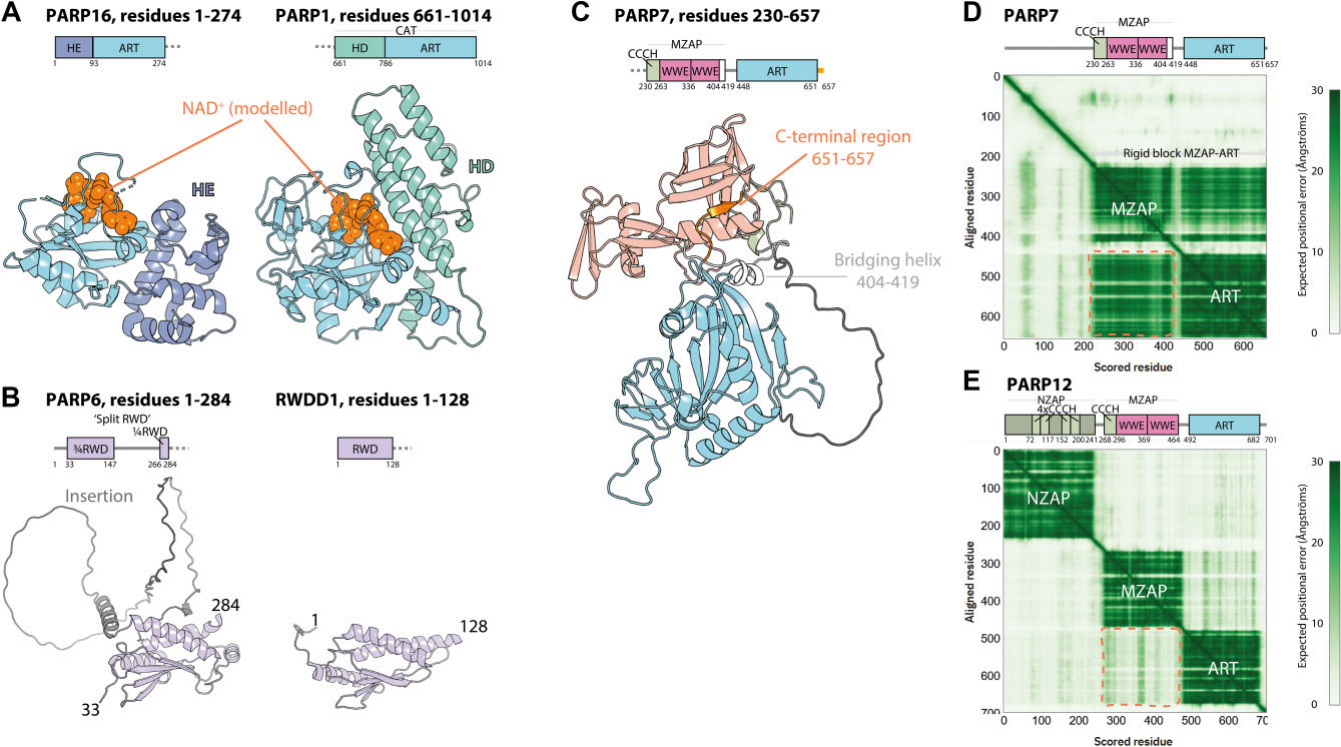
Insights into domain structures and interdomain interactions of PARP16, PARP6, PARP7 and PARP12 from experimental structures and AlphaFold2 models. (**A**) Comparison of catalytic fragments of PARP16 and PARP1. Domain architecture and crystal structures are shown (PDBs 4F0D and 1A26), coloured according to domain composition. NAD^+^ (orange spheres) was modelled in the same way as in Figure 3B. (**B**) Comparison of the AlphaFold2 model of the split RWD domain of PARP6 with the NMR structure of the RWD domain of RWDD1 (PDB 2EBM). (**C**) Fragment of the AlphaFold2 model of PARP7. Elements connecting the MZAP and ART regions into one rigid arrangement are indicated. (**D** and **E**) Domain architectures and AlphaFold2 matrices of PARP7 and PARP12. The regions on the matrix predicting the presence of MZAP:ART interactions (in PARP7) or lack of those (in PARP12) are indicated with orange dashed lines.

AF2 models offer interesting insights into clade 6 members PARP6, PARP8 and PARP16. These three PARPs share a similar helical appendage to the ART domain—previously resolved experimentally for PARP16 (71)—which, as mentioned above, we propose to call the HE to distinguish it from the distinctly different HD, as found in PARP1, PARP2, PARP3 and PARP4. Although the HE has been described as a putative regulatory extension of the catalytic ART domain (71), it does not occlude the NAD^+^-binding site in the way the HD of PARP1 does, suggesting a different mode of regulation or, perhaps more likely, a structural or a protein:protein interaction function (Figure 5A). FoldSeek did not yield any high-confidence homologues of the HE in other proteins.

In addition to the HE and ART, PARP16 contains a downstream transmembrane (TM) helix, which has been previously reported to target the protein to the endoplasmic reticulum (ER) (72), followed by a short C-terminal helix that would face the ER lumen.

In contrast, PARP6 and PARP8 do not contain the C-terminal TM helix. Instead, they are extended on the N-terminal side where they both contain a previously unannotated putative C4-type ZnF followed by a ‘split’ RWD domain (named after RING-fingers, WD proteins and DEXDc-like helicases) with a long, mostly putatively disordered, insertion (Figure 5B).

The C4-type ZnF found in PARP6 and PARP8—which we labelled ‘C4*’ in Figure 2—is structurally distinct from the third ZnF of PARP1 (labelled ‘C4’), which also has a C4 configuration. We did not find any structural homologues of this ZnF in other proteins using FoldSeek. While its function is unclear, it seems to be rigidly connected to the split RWD, so it might be functionally related to it.

In general, the RWD domain is evolutionarily and structurally related to the ubiquitin-conjugating core (UBC) domain of E2 enzymes involved in ubiquitylation and related PTM systems, but, unlike UBC, the RWD does not typically contain a conserved cysteine residue (73). RWD domains of PARP6 and PARP8 could hint at a link to the ubiquitylation, SUMOylation, or a related pathway, although examples of RWDs performing roles without a direct link to ubiquitylation are known, *e.g*. among kinetochore proteins (74). While the split RWD domains in PARP6 and PARP8 could not be detected with HHPred by analysing full-length sequences of these proteins, the sequence of the split RWD of PARP6 from which the insertion seen in the AF2 model was deleted yielded a low-confidence (E-value of 180) hit against the NMR structure of RWD domain-containing protein 3 (RWDD3), a protein identified as a binder of UBC9, the E2 for small ubiquitin-like modifier (SUMO) (75). Despite sequence divergence, the split RWDs of both PARP6 and PARP8 superpose well with canonical RWD domains from RWDD proteins over the main structural elements (RMSD of 2–3 Å over ∼70 C_alpha_ atoms). In addition to the split RWD domain, PARP8 contains a further, N-terminal RWD connected via an intrinsically disordered linker to the rest of the protein, but this domain appears severely diverged from canonical RWD domains (RMSD of ∼9 Å over ∼70 C_alpha_ atoms).

### PARP7, PARP11, PARP12 and PARP13

PARP7 (also known as TIPARP or PARPT for 2,3,7,8-tetrachlorodibenzo-*p*-dioxin (TCDD)-inducible PARP), PARP11, PARP12 and PARP13 (also known as ZAP, for ZnF antiviral protein) belong to clade 3. They make one subset of PARPs that contain the WWE domain (named after conserved amino-acid residues), a small domain with some similarity to the beta-grasp fold of ubiquitin (76). In several proteins, the WWE domain has been shown to interact with PAR chains. While the canonical WWE domain of RNF146 recognises an iso-ADP-ribose moiety (formed by parts of two consecutive ADP-ribose units in a PAR chain) (77,78), the single WWE domain of PARP11 has been reported to prefer the terminal ADP-ribose unit (79).

Unlike PARP11, which contains just one WWE domain, PARP7, PARP12, and PARP13 contain a larger compact arrangement composed of a CCCH-type ZnF and two WWE domains, which we propose to call MZAP (for middle domain of ZAP), by analogy to the NZAP domain mentioned below. The experimental structure of this region from PARP13 has very recently been determined independently by two groups (80,81), revealing a virtually identical arrangement to the AF2 models (RMSD between PDB 7KZH and the PARP13 AF2 model of 0.4 Å over 153 C_alpha_ atoms). Biochemical experiments showed that only the second WWE in MZAP is functional in recognising PAR chains, again with a preference for the terminal unit (81).

Interestingly, AF2 predicts, with high confidence, that the ART domain of PARP7 forms an inter-domain interaction with the MZAP arrangement, with the result that all structured elements of PARP7 form one compact assembly (Figure 5C), as also reflected in the AF2 matrix (Figure 5D, top). The key contacts are made by the very C-terminal region protruding from the ART domain (residues 651–657), which complements the beta-sheet of the first WWE domain and a bridging helix (residues 404–419) that is wedged between the ART and the WWE. This arrangement is not observed for PARP12 (Figure 5D, bottom) or PARP13, in which the MZAP and ART portions appear to be flexible relative to each other. We predict that the close association between different parts of PARP7 could preclude production of soluble isolated fragments corresponding to individual domains, perhaps explaining the lack of crystal structures of the ART and MZAP parts of PARP7.

On the other hand, PARP12 and PARP13—but not PARP7—contain an N-terminal domain that we labelled NZAP (for N-terminal domain of ZAP) following previous convention (82). This domain—a compact assembly of four CCCH-type ZnFs, several additional alpha-helices and a beta-sheet—has been visualised experimentally and shown to mediate RNA recognition (82,83). The large intrinsically disordered region in PARP13 might in part contribute to RNA binding and is consistent with localisation of this protein to stress granules rich in RNA and proteins (84). Similarly, the N-terminal disordered region of PARP7 might be related to its WWE domain-dependent compartmentalisation in nuclear condensates (85).

### PARP9, PARP10, PARP14 and PARP15

As mentioned in the general remarks above, among the most interesting insights provided by the AF2 models of PARPs is the apparent prevalence of previously unannotated KH domains in a subset of clade 3 PARPs comprising PARP9 (also known as B-aggressive lymphoma 1 or BAL1), PARP10, and, where there are particularly many, PARP14 (also known as BAL2). The KH domain, named after the heterogenous nuclear ribonucleoprotein (hnRNP) K in which it was first identified (86), is found in all domains of life but is particularly widespread in eukaryotes. It functions primarily as sequence-specific RNA- or, less commonly, single-stranded DNA-binding module, with a single KH domain canonically recognising four unpaired RNA bases (87). The term KH domain has been used for two similar but topologically different structural arrangements, referred to as type I and type II (88); all the instances that we predicted in PARPs are of type I.

**Figure 6.**
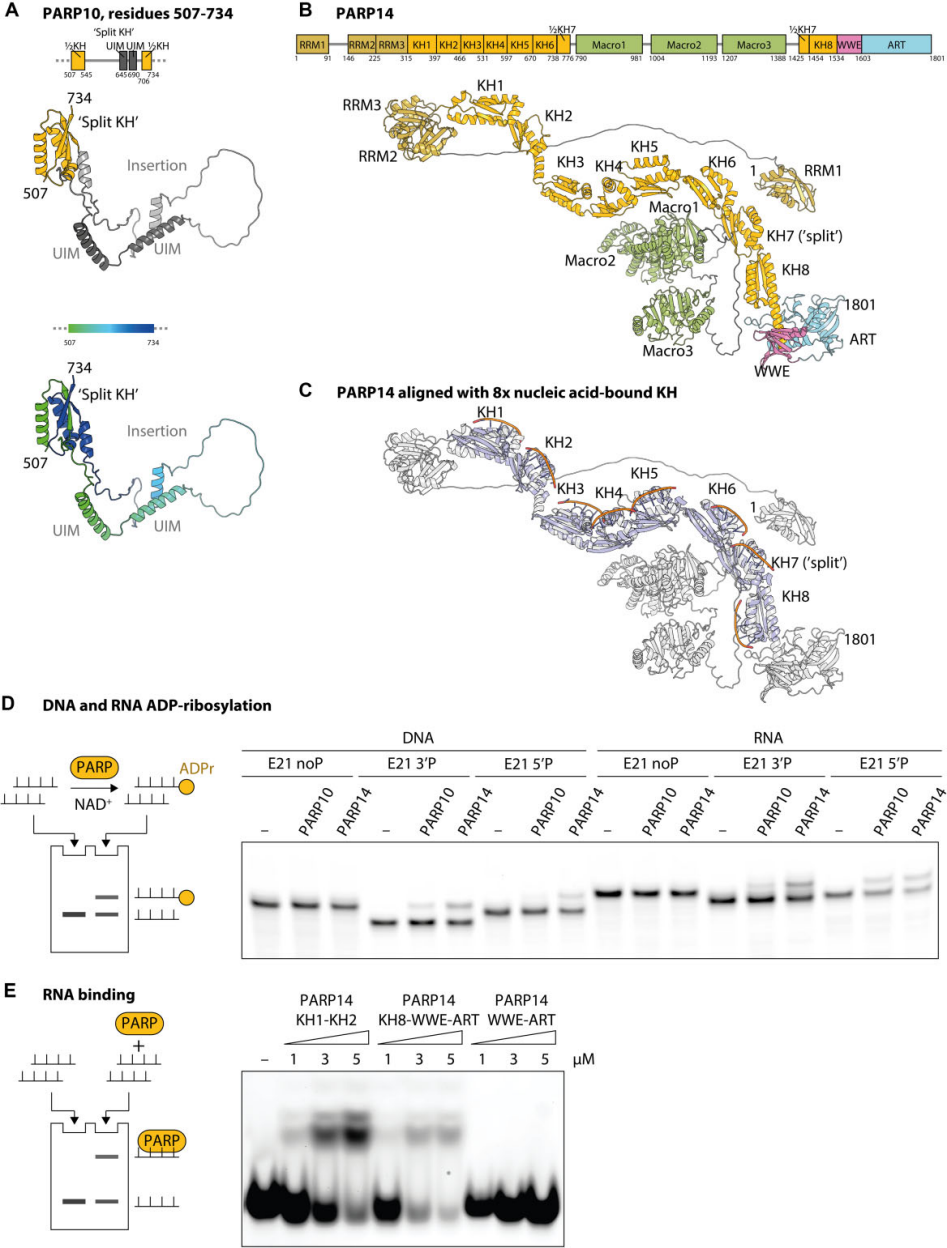
Insights into domain structures of PARP10 and PARP14 from AlphaFold2 models. (**A**) Domain architecture and AlphaFold2 model of the region corresponding to the ‘split’ KH domain of PARP10. Structural models are coloured according to domain composition (top) or residue number (bottom, according to the indicated colour scale from dark blue to dark green). (**B**) Domain architecture and AlphaFold2 model of PARP14. Structural models are coloured according to domain composition and labelled with domain names. (**C**) Structural model of PARP14 from B aligned with eight copies of the crystal structure of a KH domain of hnRNP K (light blue) bound to single-stranded DNA (brown, shown only in contact regions) (PDB 1J5K). (**D**) DNA and RNA ADP-ribosylation assay of catalytic fragments of PARP10 and PARP14. The principle of the assay is illustrated on the left and further explained in Materials and methods. The ADP-ribosylation of fluorescently-labelled single-stranded DNA or RNA oligomers without (noP) or with terminal phosphate moieties (3′P or 5′P) was monitored using gel mobility shift as a readout. (**E**) PARP14 fragments KH1–KH2 and KH8-WWE-ART but not WWE-ART bind Cy3-labelled single-stranded RNA according to an electrophoretic mobility shift assay (EMSA). A decreased total RNA amount in wells with PARP14 KH8-WWE-ART might be due to a slight nuclease contamination. Experiments shown in (D) and (E) were repeated at least three times with similar results.

In PARP9, PARP10, and PARP14, one of the KH domains is ‘split’ at the sequence level, containing a large insertion. In PARP9 and PARP14, the insertion contains previously annotated Macro domains (two in PARP9 and three in PARP14), some or all of which seem to be functional as ADP-ribose binding domains (89,90). In PARP10, in contrast, the insertion in the split KH domain contains previously reported UIMs (55), which, through simultaneous binding to two ubiquitin molecules, could potentially recognise a specific poly-Ub linkage (Figure 6A).

Between the split KH domain and the ART, there is, in each case, one more KH domain with an elongated helix that connects to the ART either through a loop or—in PARP14—through a WWE domain. The presence of this WWE domain hints at an evolutionary link between the PARPs discussed in this section and those in the previous one, as reflected in their joint classification as clade 3, but the WWE domain of PARP14 appears diverged in sequence and devoid of the PAR-binding function (79). In PARP9, PARP14, and PARP15, the ability to recognise ADP-ribose could be taken over by Macro domains.

In addition to the domains described so far, PARP10 contains a further KH domain at the C-terminal side of the split KH (making a total of three KH domains in PARP10), preceded by a long linker and the N-terminal compact arrangement of three RRM domains (for RNA recognition motif), which likely also participate in sequence-dependent RNA binding. RRM domains, which might be distantly related to KH domains, show more plasticity in terms of the type of RNA ligand that they engage (91). A rigid arrangement of three consecutive RRMs found in PARP10 might recognise a particular RNA tertiary structure.

PARP14 has a more elaborate structure than PARP9 or PARP10, with six KH domains arranged in-line upstream of the split domain (making, with the further downstream domain, eight KH domains in total), capped by two tandem RRM domains and a further, N-terminal RRM connected by a flexible linker (Figure 6B). Strikingly, the two RRM domains and eight KH domains of PARP14 are arranged one directly after another in a helical manner that could track a long RNA (or possibly single-stranded DNA) molecule, with the capping RRMs potentially recognising a particular structure at one end of the RNA. Indeed, superposing each KH domain in PARP14 with an NMR structure of a single-stranded nucleic acid-bound KH domain from another protein (92) shows that the linear assembly could recognise a long nucleic acid fragment (Figure 6C) of perhaps over 40 RNA/DNA bases, especially if one takes into account the RNA-binding potential of the RRM domains. The recognised RNA/DNA could also be shorter if not all KHs and/or RRMs are functional in nucleic acid binding. Of note, more diverged KH domains have in the past been implicated in protein:protein rather than protein:nucleic acid interaction (93–95), and the same could be the case for at least some of those predicted in this study in PARPs. More detailed analysis of surface electrostatics and conservation of important residues could shed further light on the potential RNA interaction.

We observed that some of the KH domains predicted here can be detected with HHPRED, but that has never been reported. Most of the predicted KH domains, including the split ones, superpose well with experimental structures of canonical type I KH domains (RMSD of 1.5–3 Å over 50–60 core C_alpha_ atoms when comparing KHs from PARP AF2 models individually with PDB 1J5K), but some, e.g. KH1 of PARP14, are more diverged structurally (RMSD of ∼6 Å over ∼60 C_alpha_ atoms).

The large numbers of putative RNA-interacting modules revealed are consistent with reports that implicate PARP9 and/or PARP14 in RNA-binding and anti-viral activity (66,96–100). For PARP10, while a biological link to RNA or single-stranded DNA is unknown, an RNA ADP-ribosylation activity—i.e. attachment of ADP-ribose to RNA via its terminal phosphate groups—has been reported (101–103). It remains to be established if these PARPs bind RNA and, if so, whether the principal function of RNA binding is to recruit specific substrates for RNA ADP-ribosylation and what role such a ‘post-transcriptional modification’ of RNA could play.

Finally, the AF2 model of PARP15 (also known as BAL3), the diverged clade 3 member only present in humans and related species (58), confirms a previous annotation of two Macro domains followed by ART (Figure 1). The three domains are predicted to be flexible relative to each other. PARP15 does not have any putative RNA-binding domains.

### *In vitro* DNA and RNA ADP-ribosylation activity and RNA binding capability of PARP14

The predicted domain architecture of PARP14 could suggest that it specifically recognises and possibly ADP-ribosylates nucleic acid substrates. As PARP14 has never been shown to be able to modify nucleic acids, we examined this putative activity experimentally using an *in vitro* assay. Since we could not purify the full-length enzyme, we focused on its extended catalytic fragment that encompasses WWE and ART domains (residues 1459–1801). Our experiments showed that while PARP14 cannot ADP-ribosylate model nucleic acid substrates with unmodified ends, it efficiently catalyses ADP-ribosylation of single-stranded RNA and DNA molecules with a phosphate at the termini (Figure 6D). This activity resembles that of PARP10 (101).

Since we expect the predicted binding domains in PARP14 to target this activity to specific substrate(s), we attempted recombinant production of fragments of PARP14 that include at least some predicted KH domains. Two such fragments, encompassing KH1-KH2 (residues 316–468) or KH8-WWE-ART (residues 1453–1602), were produced in a pure recombinant form and showed an efficient binding at micromolar concentrations to an RNA probe in an electrophoretic mobility shift assay (EMSA). A catalytic fragment of PARP14, WWE-ART, introduced above was not able to interact with RNA under the same conditions.

Overall, the successful detection of nucleic acid binding and ADP-ribosylation activity for PARP14 fragments demonstrates the usefulness of AF2-driven domain annotation for inferring molecular function.

### The PARP family expanding? LRCC9, TASOR and NEURL4

The PARP family is defined as a group of proteins that contain an ART domain similar to that in the founding member PARP1. We wondered whether the question of family membership could be revisited in the light of new opportunities offered by AF2-mediated structure prediction. We therefore used FoldSeek to search all available AF2 models (the AlphaFold/Proteome v4 collection) for human proteins with regions that are structurally highly similar to PARP1’s ART domain. As a search model, we used a previously published crystal structure (60) (PDB 6BHV).

This analysis uncovered 19 hits with very high confidence (*E*-value of < 10^−5^), which include 17 canonical human PARPs discussed above as well as two additional proteins, LRRC9 and TASOR. Both of these proteins are known to be related to the PARP family (104,105), but what the FoldSeek result additionally suggests is that their ART domains are more similar to the ART domain of PARP than to those of some canonical PARPs, at least at the level of local structure as probed by the 3Di/AA algorithm. This could argue for the inclusion of LRRC9 and TASOR in the PARP classification, despite divergence from PARP1 itself and possible lack of catalytic activity (104,105).

Additionally, the FoldSeek analysis identified one further hit, NEURL4, with a lower, but still high, confidence (*E*-value of around 10^−2^); however, in this case the local structural similarity with PARP1’s ART domain is lower than for all canonical PARPs. NEURL4 is known to be related to PARPs (105–107) and our analysis justifies its description as ‘PARP-related’ or ‘PARP-like’, but arguably not its inclusion in the PARP protein family.

The FoldSeek search did not identify two other human proteins that in the past have been suggested to contain putative ART domains similar to those in TASOR: TASOR2 and TEX15 (105). In the first case, upon inspection of the precalculated AF2 model of TASOR2 and additional bioinformatic analysis, we believe that this protein does not in fact contain a complete domain similar to ART. In the case of TEX15, the extreme sequence length (2789 residues) means that there is no precalculated AF2 model available in the AF Protein Structure Database for FoldSeek to scan. Thus, FoldSeek could not have detected structural similarity between PARP1’s ART and TEX15, even though a brief analysis appears to confirm the previous annotation of a TASOR-like ART domain within TEX15.

Overall, despite certain limitations, quantitative insights into the predicted local structural similarity of ART domains in PARPs and PARP-like proteins offered by AF2 and FoldSeek could be used as an approach to better define the boundaries of the PARP protein family. However, prior to such attempts, there needs to be a community debate on which criteria should be used to define PARP family membership.

## DISCUSSION

The development of the AlphaFold2 AI-based protein structure prediction tool has rapidly revolutionised structural biology. Among other uses, high accuracy prediction offers unprecedented access to the domain architecture of protein families, expanding existing annotations that are based primarily on sequence motif analysis.

Here, we applied AF2 to better characterise domain architecture of the 17 human members of the PARP protein family, defined by the presence of a PARP1-like ART domain. In most cases, PARPs are catalytically active in protein MARylation or PARylation. While the PARP family has long been known to be particularly diverse in its domain composition, the domain annotation had remained incomplete. We believe that our predictions finalise this task, offering what is likely to be an essentially complete annotation of structured domains and long intrinsically disordered regions (Figure 2). We have limited ourselves to canonical PARP members, but we note that AF2 structural models could be used as the basis for redefining the PARP family to include related proteins that are diverged on the sequence level but sufficiently similar on the structure level. Above, we include a preliminary analysis of this kind.

While some (but not all) instances of the domains annotated here for the first time could have been detected with sequence-based techniques such as HHPred, AF2—combined with visual inspection and structural search and alignment—offers a surer way of identifying low homology and defining new domain types. Interestingly, AF2, possibly owing to its use of computational attention mechanisms and transformers that detect long-range inter-residue relationships (42), efficiently predicts structures of split domains composed of two parts that are not consecutive in sequence. Such ‘splitting’ poses a problem for sequenced-based methods, as illustrated above for the case of split RWD domains, which could only be detected with HHPred once the sequence of the insertion seen in the AF2 model was deleted.

While the newly annotated domains and other insights should be validated experimentally, the structures of all annotated regions were predicted with high accuracy (according to validated AF2 criteria (40,51)) and produced convincing models. In most cases, these models overlapped well with known domains, allowing confident domain identification. Analysing AF2 matrices in addition to models allows prediction of larger rigid arrangements stabilised by inter-domain contacts. The approach used above could be extended by combining it with an evolutionary analysis of PARP domain composition across species, or with mapping sequence conservation across evolution upon structural models; the latter task can be easily performed using the ConSurf server, as we illustrate for the MVPID domain of PARP4 (Figure 4D).

As its main insights, our study i) predicts several previously undetected putatively RNA- or DNA-binding KH domains in PARP9, PARP10 and PARP14, ii) predicts E2-related RWD domains in PARP6 and PARP8, and iii) suggests a high degree of structural homology between parts of PARP4 and PARP1. High incidence of probable nucleic acid-binding domains in some PARPs could suggest recognition of specific long DNA or RNA ligands in a manner akin to that proposed for other proteins rich in domains of these types (108). Overall, the analysis strengthens the known links between ADP-ribosylation and RNA biology and ubiquitylation, while offering specific new insights into each PARP subgroup.

Of particular interest is PARP14, which is predicted to have a linear arrangement of multiple RNA- or DNA-binding domains that could recognise a specific, long, single-stranded nucleic acid fragment (Figure 6B and C). Prompted by this observation, we examined *in vitro* properties of fragments of PARP14, demonstrating the ability of KH domain-containing fragments to bind RNA and that of a catalytic fragment to ADP-ribosylate terminally-phosphorylated DNA or RNA fragments (Figure 6D and E). While no *in-vivo* PARP14 substrates are yet known, PARP14 has been reported to play an important role in the immune response against viral infections, including those caused by coronaviruses (109). We have previously shown that the recombinant coronavirus macrodomain protein (part of Nsp3) can reverse PARP14 automodification, suggesting that the two proteins form a pair of mutually opposed activities as part of the virus-host rivalry (110). Notably, another coronaviral protein, Nsp15, cleaves viral RNA molecules to prevent activation of host RNA sensors (111–114). The Nsp15-catalysed cleavage generates RNA fragments that have phosphorylated ends, making them amenable to PARP14’s activity revealed in our study. PARP14-mediated recognition and ADP-ribosylation of RNA could serve as a defence mechanism, preventing viral RNA from evading the host's immune response. In addition, given the involvement of PARP14 in repair of stalled replication forks and possibly other DNA repair pathways (115), its apparent DNA ADP-ribosylation activity could also be relevant *in vivo*. Future studies should address these questions.

Our analysis suggests that PARP proteins are mostly structured, with longer (>100 amino-acid residues) intrinsically disordered regions found in PARP4, TNKS1/PARP5A, PARP6, PARP7, PARP8, PARP10 and PARP13. The presence of such regions—parts of which might become ordered upon binding to interaction partners—is likely functionally important and could be related to condensate formation (116,117). Disordered regions of various length could also harbour short linear motifs (SLiMs), including PTM sites (117,118). In addition to predicting structures of individual domains and proteins, AF2 can predict structures of some protein complexes and thus could be used to model PARP oligomerisation and interactions between PARPs and their binding partners. As prediction of complexes is, on average, less accurate than that of folds of individual proteins, it will be particularly important to verify such models experimentally. Moreover, experiments can provide parameters such as equilibrium dissociation constant (*K*_d_) and association/dissociation rate constants (*k*_on_/*k*_off_) that cannot be reliably obtained through computational approaches but are necessary for evaluating the functional importance of protein:protein interactions. Finally, even though AF2 can be used to predict interactions with large numbers of proteins in an automated manner—as in the new AlphaPulldown pipeline (119)—experimental approaches to identifying binding partners in cells or extracts are still likely to be more efficient at finding new, unexpected connections. Recent studies show that binding partners can have a dramatic influence on PARP function to the point of changing their catalytic properties and substrate specificity, as in the case of HPF1-dependent regulation of PARP1 and PARP2 (31,120–123). It is pertinent to ask if any other members of the PARP family are perhaps similarly regulated by so far unknown regulatory interactors.

The validated high accuracy of AF2 predictions offers an unprecedented perspective on the structure-function relationship in proteins. However, it is far from providing an exhaustive description of the analysed systems. The well-studied cases of PARP1, PARP2 and PARP3, on the one hand, and tankyrases, on the other, teach us that structural models—even if very accurate—do not fully explain function. More specifically, the case of PARP1 and its closest homologues demonstrates that protein domains are not static. Our new NMR data (Figure 3B and C) and previous studies (59,124–126) demonstrate that these PARPs can sample various conformations, with domains either mobile or rigid with respect to each and either structurally stabilised or partially unfolded, all of which can be of major importance for function, at least for highly dynamic, allosteric systems such as PARPs. The case of tankyrases, on the other hand, serves as a reminder of the importance of noncovalent protein oligomerisation or polymerisation (39). AF2 could potentially be used to predict both conformational diversity and multimerisation of PARP proteins, but this is likely to be less accurate than predicting a dominant state of a single protomer and, in the case of multimerisation, it would be computationally expensive for large PARPs.

One aspect of protein function into which AF2 or related programmes do not (so far) offer a direct insight are interactions with non-protein factors such as—in the case of PARPs—nucleic acids (DNA and RNA), PAR chains, or small molecules (ADP-ribose, NAD^+^, inhibitors, etc.). Such interactions can be proposed based on predicting homology to other domains known to mediate them—as we have done in this study. Moreover, further insight might be gained by analysing conservation of pockets and interfaces necessary for interactions. Ultimately, however, binding to nonprotein ligands must be verified and characterised experimentally. Experiments can also detect changes in protein conformation upon ligand binding, as illustrated in our NMR analysis of PARP1 bound to a DNA break (Figure 3B).

Finally, it is important to mention that as AF2 and related programmes work by detecting patterns in protein structures and sequences—and not by simulating physicochemical forces at play in protein folding (except at the final, model relaxation step)—it is possible that in the future they could be supplemented by molecular dynamics and related computational methods that take into account protein physics.

We propose the above analysis of the PARP family as a springboard for further experimental investigations and modelling efforts that aim at elucidating the biological roles of the PARP-family members. Similar comprehensive analyses of structural models of other protein families could also aid their investigation.

## Supplementary Material

gkad514_Supplemental_File

## Data Availability

NMR chemical shift assignment data for human PARP1 has been deposited at the BMRB under the following accession codes: ^1^H, ^13^C and ^15^N backbone assignments for BRCT domain, 51892; partial ^1^H and ^15^N backbone assignments for full-length free protein, 51893; partial ^1^H and ^15^N backbone assignments for DNA-bound full-length protein, 51894.

## Notes

Present address: Laura E. Easton, Merck Life Sciences, Stirling University Innovation Park, Stirling FK9 4NF, UK.

Present address: Wing-Fung Wu, HiFiBiO (HK) Limited, Unit 517, 5/F, BiotechCentre 2, Phase 2, 11W Hong Kong Science and Technology Park, Shatin, NT, Hong Kong, China.
