# Supplementary material for: Updated protein domain annotation of the PARP protein family sheds new light on biological function
Source: Nucleic Acids Res. 2023 Jun 16;51(15):8217–36. doi: 10.1093/nar/gkad514 (PMC10450202; doi:10.1093/nar/gkad514)
Supplement: gkad514_Supplemental_File [file gkad514_supplemental_file.pdf]

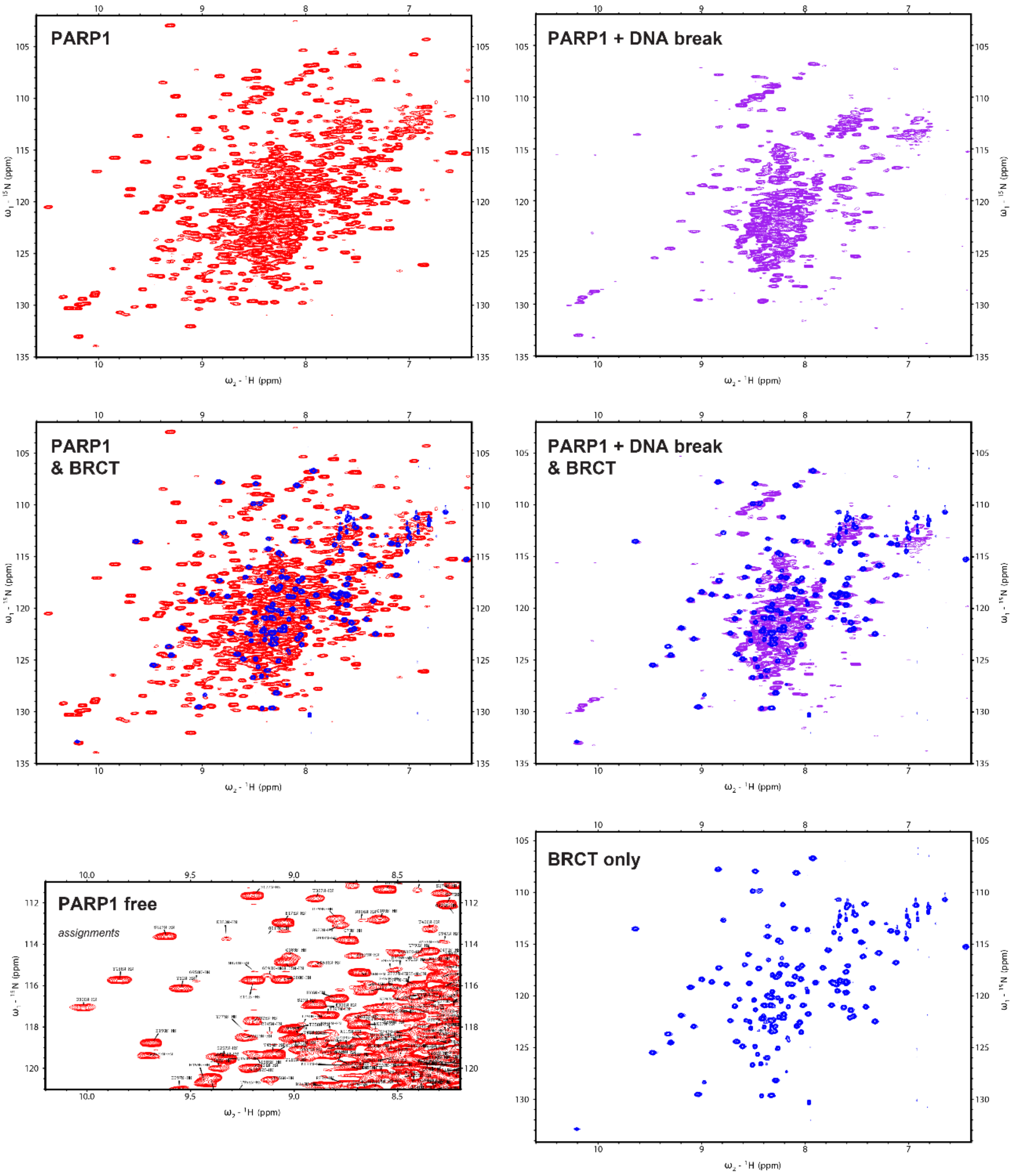

Supplementary Figure 1. NMR analysis of PARP1

$[^{15}N,^{1}H]$-TROSY spectra of full-length PARP1 with (purple) and without (red) a DNA dumbbell mimicking a single-stranded DNA break compared to the spectrum (blue) of an isolated BRCT domain. The middle two panels show overlays of the spectra shown in the top two panels with the spectrum of isolated BRCT, to emphasise the extent of similarity in the corresponding dispersed signals. The bottom left panel shows a region of the spectrum of the full-length free PARP1 illustrating the extent of crosspeak assignments. Relative to the HSQC spectrum, TROSY spectra are shown displaced by 46 Hz (= $^{1}J(^{15}N,^{1}H)/2$) downfield in the ^{1}H frequency dimension and 46 Hz upfield in the ^{15}N frequency dimension, in order to remove the expected systematic offset between the two types of spectrum.
